# Targeting the Leloir Pathway with Galactose-Based Antimetabolites in Glioblastoma

**DOI:** 10.3390/cancers16203510

**Published:** 2024-10-17

**Authors:** Martyn A. Sharpe, Omkar B. Ijare, Sudhir Raghavan, Alexandra M. Baskin, Brianna N. Baskin, David S. Baskin

**Affiliations:** 1 Kenneth R. Peak Brain and Pituitary Tumor Treatment Center, Houston Methodist Hospital, Houston, TX 77030, USA; sudhir.raghavan@saleseer.com (S.R.); ambaskin@houstonmethodist.org (A.M.B.); baskinb1@gator.uhd.edu (B.N.B.); dbaskin@houstonmethodist.org (D.S.B.); 2 Department of Neurosurgery, Houston Methodist Neurological Institute, Houston Methodist Hospital and Research Institute, Houston, TX 77030, USA; 3 Houston Methodist Academic Institute, Houston, TX 77030, USA; 4 Weill Cornell Medical College, Cornell University, New York, NY 10065, USA; 5 Texas A & M Medical School, Houston, TX 77030, USA

**Keywords:** antimetabolite, fluorogalactose, galactose scavenging, glioblastoma, Leloir pathway

## Abstract

Background: Glioblastoma (GBM) uses Glut3 and/or Glut14 and the Leloir pathway to catabolize D-Galactose (Gal). UDP-4-deoxy-4-fluorogalactose (UDP-4DFG) is a potent inhibitor of the two key enzymes, UDP-galactose-4-epimerase (GALE) and UDP-Glucose 6-dehydrogenase (UGDH), involved in Gal metabolism and in glycan synthesis. The Gal antimetabolite 4-deoxy-4-fluorogalactose (4DFG) is a good substrate for Glut3/Glut14 and acts as a potent glioma chemotherapeutic. Methods: Primary GBM cell cultures were used to examine toxicity and alterations in glycan composition via lectin binding in fixed cells and by Western blots. Toxicity/efficacy in vivo data was performed in mouse flank and intracranial models. The effect of 4DFG on D-glucose (Glc) metabolism in GBM cells was assessed by using ^13^C NMR-based tracer studies. Results: 4DFG is moderately potent against GBM cells (IC_50_: 125–300 µM). GBM glycosylation is disrupted by 4DFG. Survival analysis in an intracranial mouse model showed that treatment with 4DFG (6 × 25 mg/kg of 4DFG, intravenously) improved outcomes by three-fold (*p* < 0.01). Metabolic flux analysis revealed that both glycolytic and mitochondrial metabolic fluxes of [U-^13^C]Glc were significantly decreased in the presence of 4DFG in GBM cells. Conclusion: A functional Gal-scavenging pathway in GBM allows Gal-based antimetabolites to act as chemotherapeutics. 4DFG is metabolized by GBM in vitro and in vivo, is lethal to GBM tumors, and is well tolerated in mice.

## Simple Summary

Glioblastoma (GBM) uses the Leloir pathway to catabolize D-Galactose (Gal) for tumor growth. Selective targeting of the Leloir pathway with Gal-based antimetabolites has potential for the treatment of GBM. Here, we tested the effect of a Gal-based antimetabolite, 4-deoxy-4-fluorogalactose (4DFG) on the viability and metabolism of GBM cells in culture. 4DFG is a good Glut3/Glut14 substrate and acts as a potent glioma chemotherapeutic. GBM cell cultures were used to examine toxicity and alterations in glycan composition. 4DFG is moderately potent against GBM cells in vitro (IC_50_: 125–300 µM). Glycosylation in GBM was disrupted by 4DFG. The effect of 4DFG on D-glucose (Glc) metabolism in GBM cells was assessed by using ^13^C NMR-based tracer studies. ^13^C-NMR-based metabolic flux analysis revealed that both glycolytic and mitochondrial metabolic fluxes of [U-^13^C]Glc were significantly decreased in the presence of 4DFG in GBM cells. Survival analysis in an intracranial mouse model during treatment with 4DFG (6 × 25 mg/kg of 4DFG, intravenously) showed improved outcomes by three-fold (*p* < 0.01). 4DFG is metabolized by GBM in vitro and in vivo, and is lethal to GBM tumors, but well tolerated in mice. A functional Gal-scavenging pathway in GBM allows Gal-based antimetabolites to act as chemotherapeutics.

## 1. Introduction

**Figure 1:**
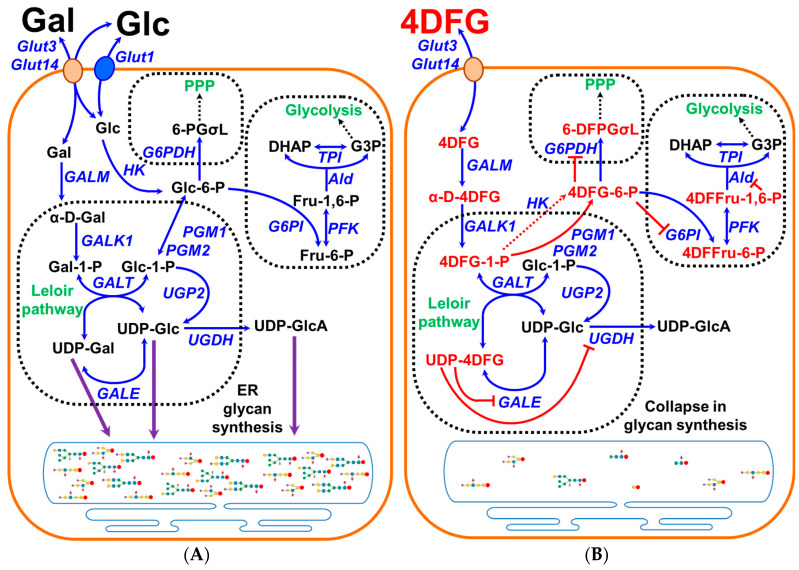
Graphical representation of the scavenging of galactose in cancer cells and the effects of 4DFG. (**A**) shows the schematic representation of galactose (Gal) metabolism in cancer cells. Gal is imported into the cells by the transporters Glut3/Glut14, and then by the action of the Leloir pathway enzymes (GALM, GALK1, GALT and GALE) it get converted into glucose-1-phosphate (Glc-1-P) and can be used to generate glycan precursors, UDP-Glc, UDP-Gal and uridine diphosphate glucuronic acid (UDP-GlcA), by the action of enzymes UGP2/GALE/UGDH) or it gets converted into glucose-6-phosphate (Glc-6-P) and further metabolized by glycolysis or the pentose phosphate pathway (PPP). (**B**) schematically shows the import and metabolism of 4-deoxy-4-fluoro-galactose (4DFG) to generate 4DFG metabolites (4DFG-6-P and UDP-4DFG) and their inhibitory action on the glycan synthesis and the inhibition of both glycolysis and the PPP metabolic pathways.

Glioblastoma (GBM) is the most common primary brain tumor with an incidence rate of 3.27 per 100,000 and a median survival of only 8 months following diagnosis [1]. Although the standard of care for the treatment of GBM with surgery, radiation and chemotherapy has shown some survival benefit (~16 months), the quality of life remains very poor [2]. In some adult mammalian cells (for example, liver, kidney and gut) which express Leloir pathway enzymes, a galactose (Gal) scavenging pathway can operate [3]. The Leloir pathway allows these cells to utilize Gal as a catabolic substrate and Gal acts as a precursor for the synthesis of UDP-sugars to be utilized in glycan synthesis or to power glycolysis and the pentose phosphate pathway (PPP) [4,5], schematically shown in Figure 1A. Once transported inside the Leloir-competent cell, β-D-Gal gets converted to α-D-Gal by the enzyme galactose mutarotase (GALM). The key kinetic step in Gal metabolism is metabolic trapping of α-D-Gal by galactokinase (GALK1), which generates galactose-1-phosphate (Gal-1-P). Later, Gal-1-P gets uridinated to uridine diphosphate galactose (UDP-Gal) by Gal-1-P uridylyltransferase (GALT). The UDP-galactose epimerase (GALE) acts as a UDP-Gal/UDP-Glc ‘toggle’, oxidizing and then re-reducing the 4′-OH of these uridinated sugars allowing their interconversion. The UDP-Glc formed is converted into Glc-1-P and then into Glc-6-P by phosphoglucomutases (PGM1 and PGM2). This is the essence of the Leloir pathway, the overall conversion of Gal and ATP to Glc-6-P and ADP, allowing carbons from Gal to enter the glycolysis and the pentose phosphate pathway (PPP). As all nucleated cells generate glycans, many of the enzymes of the Leloir pathway have dual functionality as key enzymes in glycan synthesis, GALT, GALE and PGM1/2, and are ubiquitously expressed.

Like fetal/infant hepatocytes, GBMs are highly efficient in catabolizing Gal [5], and may be linked with the expression of fetal-liver proteins in GBM [6], and this could be a result of the general property of oncofetal reprogramming in cancer [7]. Upregulation of enzymes of Gal metabolism has been observed in aggressive GBMs and Gal metabolism has been identified as one of the five metabolic pathways in the GBM biomarker panel [8]. The Gal metabolic pathway has also been found to be utilized by metastatic skin cutaneous melanoma [9] and breast cancer [10]. Highly aggressive breast cancer cells upregulate the enzymes of the Leloir pathway when presented with galactose, in vivo [10]. The ability of cells to efficiently transport either Glc or Gal is unique to Glut3/14, and results from the nature of the hexose binding site of these two transporters [11,12,13,14]. The crystal structure of Glut3 bound to Glc demonstrates that the hexose 4′OH projects into the bulk aqueous phase [15]. As Glc and Gal are epimers differing only in the configuration at C-4, the hexose binding site of Glut3 and/or Glut14 accommodates either of these sugars. This contrasts with other Glut transporters, which transport Gal poorly, because the 4′OH position of the hexose is an integral part of the hydrogen bonding network between the transporter substrate pocket and the sugar.

It has been long recognized that the glycome and glycoproteome are altered in several cancers and with these changes in glycosylation, tumors drive metastatic properties, immune modulation, inhibition of apoptosis and resistance to chemotherapy [16,17]. Glycosyltransferase gene expression profiles have been used for the classification of cancers and the development of prognostic models [18]. For instance, malignant astrocytic tumors have been reported to show increased expression of highly sulfated keratin sulfate [19]. Specific terminal sialylation and fucosylation patterns of complex N-glycans have been implicated in aggressive behavior of gliomas [20]. Recently, an in vivo study using an orthotopic glioma mouse model has indicated that gliomas overexpress truncated O-linked glycans [21].

PET imaging studies with 4-deoxy-4-[^18^F]fluoro-Glc show that this tracer crosses the blood–brain barrier and accumulates in Glut3-rich cells [22]. This observation raised the possibility that 4-deoxy-4-X-Gal mimetics could be used as antimetabolite drugs in cancer cells such as GBM that express Glut3/14 and the enzymes of the Leloir pathway [5]. GBM should efficiently import 4-dexoy-4-fluoro-galcatose (4DFG), via Glut3/Glut14, and then metabolize it into UDP-4-deoxy-4-fluorogalactose (UDP-4DFG). UDP-4DFG is a potent inhibitor of the two key enzymes: UDP-galactose-4-epimerase (GALE) and UDP-glucose 6-dehydrogenase (UGDH) involved in Gal metabolism and in glycan synthesis, as depicted in Figure 1B.

We elected to use 4DFG as our lead compound for this preliminary study. Here, we investigate the effect of 4DFG on cell viability, glycan synthesis and cellular metabolism in patient-derived GBM cells. Moreover, we also tested the effect of 4DFG on tumor growth and survival in flank and intracranial mouse models.

## 2. Materials and Methods

### 2.1. Chemicals

All chemicals, reagents and antibodies were obtained from Millipore Sigma (Miamisburg, OH, USA/St. Louis, MO, USA). Uniformly ^13^C-labeled D-glucose ([U-^13^C]Glc) was purchased from Millipore Sigma (Miamisburg, OH, USA/St. Louis, MO, USA). Mice were obtained from Charles River Laboratories, Inc. (Wilmington, MA, USA). FITC-labeled lectins were from EY Laboratories (San Mateo, CA, USA).

### 2.2. Tumor and Normal Human Astrocyte Cell Lines

GBM tumor tissue specimens were harvested at the time of surgical excision and were given a laboratory ID (GBM157 and GBM175), and cell lines were generated as reported in our recent study [5]. The primary stocks of the patient-derived cells (GBM157 and GBM175) were frozen at the fourth or fifth passage and cells from these passages were used for this study.

Normal human astrocytes (NHAs) were used as control brain cell line and allowed us to make a comparison to detect aberrant glycan expression in GBM cells. NHAs were obtained from Lonza (Walkersville, MD, USA) and cultured in Astrocyte Basal Medium (Lonza) as detailed in our earlier study [5].

### 2.3. Synthesis of 4-Deoxy-4-Fluoro-Galactose (4DFG)

**Scheme 1:**
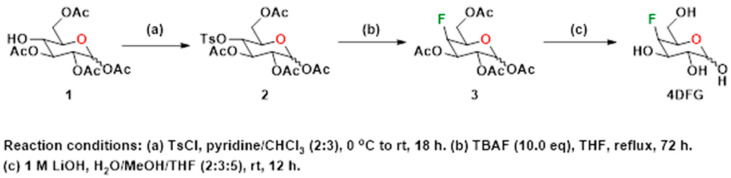
Synthesis of 4-deoxy-4-fluoro-D-galactopyranose (4DFG).

4-Deoxy-4-fluoro-D-galactopyranose (4DFG) was synthesized following the literature method [23]. Various steps involved in the synthesis of the target compound, 4DFG have been provided in Scheme 1. The 4-hydroxyl group of commercially obtained Compound **1** was converted into its corresponding p-tosyl derivative, **2**. Compound **2** was subjected to nucleophilic fluorination using tetra-n-butylammonium fluoride (TBAF) in THF under reflux conditions followed by deacetylation under basic conditions to provide the target compound 4DFG.

4-O-(4-toluenesulfonyl)-2,3,5,6-tetraacetyl-D-glucopyranose (**2**)

To a stirred solution of 2,3,5,6-tetraacetyl-D-glucopyranose, **1** (1.5 g, 4.31 mmol, 1.0 eq) in pyridine (8 mL) at 0 °C, a solution of TsCl (1.64 g, 8.62 mmol, 2.0 eq) in pyridine/chloroform (15 mL, 2:3 *v*/*v*) was added. The mixture was allowed to warm to room temperature and then stirred at room temperature for 18 h. The reaction was quenched with water (15 mL) and extracted with dichloromethane (2 × 30 mL). The combined organic phases were washed with an aqueous sulfuric acid (10% solution) (4 × 15 mL), washed with brine (1 × 10 mL), dried over anhydrous sodium sulfate, filtered and concentrated under reduced pressure. The white foamy compound **2** obtained was used for the next step without further purification. ESI MS (+ve ion mode): 503.6 (M + H), 525.5 (M + Na).

4-Fluoro-2,3,5,6-tetraacetyl-D-galactopyranose (**3**)

Compound **2** (1.0 g, 1.99 mmol, 1.0 eq) was added to a 1M solution of tetrabutylammonium fluoride (TBAF) in THF (20 mL, 19.9 mmol, 10 eq) and the mixture heated to reflux for 72 h. The reaction mixture was cooled, and then water (15 mL) was added to the mixture. The combined mixture was extracted with dichloromethane (3 × 20 mL) and the combined organic fractions were sequentially washed with saturated sodium bicarbonate (1 × 20 mL) and brine (1 × 20 mL), dried over anhydrous sodium sulfate, filtered and concentrated under reduced pressure. The crude product was purified by flash chromatography (silica gel, 2:3 hexanes: ethyl acetate, Rf = 0.48) to give **3,** as a white compound. ESI MS (+ve ion mode): 373.2 (M + Na).

4-deoxy-4-fluoro-D-galactopyranose (4DFG)

Deprotection of the acetyl groups of **3** was performed as described below. To a stirred solution of compound **3** (500 mg, 1.42 mmol, 1 eq) in 15 mL of a mixture of water/MeOH/THF (2:3:5), an aqueous 1M LiOH solution (14 mL, 10 eq) was added. The mixture was stirred at room temperature for 12 h. The reaction was then neutralized to pH 7 with acidic resin, filtered and concentrated under reduced pressure to provide a crude compound, 4DFG, that was purified using flash column chromatography (silica gel, water/*i*PrOH/dichloromethane/acetonitrile, 5:15:30:50). ESI MS (+ve ion mode): 183.3 (M + H), 205.4 (M + Na).

### 2.4. Quantification of Glut3, Glut14, GALE, GALK1 and UGDH in Glioma Tissue Microarray by Immunohistochemistry

We examined the levels of Glut3/Glut14 and key enzymes of the Leloir pathway in a group of primary glioma samples in a tumor tissue microarray, previously reported for an investigation into the factors driving monoamine oxidase B expression [24]. The tissue microarray has 8 low-grade gliomas and 21 GBM cores, and an internal control of normal human temporal lobe. In this study, the wax block was sliced into 5 μm sections, and these slices were affixed to slides and dried. The resulting slides were then dewaxed four times in xylene, twice in isopropanol, and finally rehydrated with graded ethanol. The slides were washed and made permeable using Phosphate-Buffered Saline (PBS, Thermo Fisher Scientific, Waltham, MA, USA) containing 0.1% Triton X-100. They were then heated in sodium citrate buffer (100 mM, pH 6.0) at <100 °C in a steamer for 30 min and allowed to slowly cool, for retrieval of the epitopes. Endogenous peroxidase activity was attenuated using mild conditions: 1.8% H_2_O_2_ for 5 min, 1% periodate for 5 min, 0.02% NaBH_4_ for 2 min followed by washing in PBS. The slides were blocked using a serum-free protein block (Dako North America, Inc., Carpinteria, CA, USA) and incubated with a 1:100 dilution of primary antibodies overnight at room temperature. The Dako DAB chromogen kit was used to perform visualization after HRP conjugation to the primary antibody with the HiDefTM HRP-polymer system (Cell Marque, Rocklin, CA, USA). Glut3, Glut14 and Leloir pathway enzymes were identified using rabbit primary antibodies that were obtained from Millipore Sigma: anti-Glut3 (ab15311), anti-Glut14 (HPA006539), anti-GALE (PA5-52423), anti-GALK1 (PA5-52385), anti-UGDH (PA5-28170) and anti-SLC35D2 (PA5-64148).

### 2.5. Cell Viability and Growth Studies

Studies were performed to test the effect of 4DFG on GBM cell growth and proliferation, using two primary GBM cell cultures (GBM157 and GBM175). These two primary GBM cell lines were used in our previous study focused on galactose metabolism, where we showed that galactose transport in GBM157 cells was performed mostly by Glut3, whereas in GBM175 the major transporter was Glut14 [5]. GBM157 and GBM175 were plated in 96-well plates at 1500–3000 cells per well in 0.2 mL high glucose DMEM (supplemented with 10% FBS, 1% Penicillin and Streptomycin and 2 mM L-glutamine). Twenty-four hours after seeding, the media were replaced with fresh media containing the 4DFG at various concentrations (0–300 µM). After 24 and 48 h of treatment with 4DFG, cell viability was evaluated using the fluorescent dye Hoechst 33342 at 5 µg/mL (#H3570, Thermo Fisher Scientific, Waltham, MA, USA). Cells were incubated with Hoechst 33342 for 20 min at 37 °C humidified atmosphere of air/CO_2_ (95:5, *v*/*v*) and fixed with paraformaldehyde and images were acquired using the epifluorescence microscope Nikon Eclipse TE2000-E.

### 2.6. Fluorescence Microscopy

Imaging was performed using a Nikon Eclipse TE2000-E (at 4×, 20× or 30× magnification) with a CoolSnap ES digital camera system (Roper Scientific, Trenton, NJ, USA) containing a CCD-1300-Y/HS 1392-1040 imaging array that is cooled by Peltier. Images were recorded and analyzed using Nikon NIS-Elements software (Elements 3.22.11). All images were saved as JPEG2000 files using Nikon NIS-Elements. The emission of Hoechst 33342 was collected using ex 325–375 nm, em 435–485 nm. Cell counts were performed on images taken of the center field at a magnification of 20×. Dead/dying cells were identified as having condensed nuclei with signal intensities over threefold that of the median cell nuclei or being identified as fragmented. The NIS-Elements software was also used to measure the area of cell nuclei, labeled with Hoechst 33342.

FITC-labeled lectin levels were visualized with ex 450–490 nm, em 500–550 nm at a magnification of 20×. GALE protein levels were visualized using a mouse monoclonal IgG (C-4: sc-390407, Santa Cruz Biotechnology, Dallas, TX, USA) as a primary and an Alexa-Fluor^TM^ 594 Donkey anti-mouse IgG as a secondary (A-21203, Thermo Fisher Scientific, Waltham, MA, USA) antibody at a magnification of 20×. Levels of GALE per cell were quantified by measuring total GALE fluorescence intensity in a given region of interest (ROI) which was then divided by the number of cells/nuclei in that ROI.

### 2.7. Blot Analysis

GBM cells treated with and without 4DFG were lysed in RIPA buffer containing protease inhibitor. Proteins were separated by SDS-PAGE electrophoresis. Proteins from gel were transferred to polyvinylidene fluoride (PVDF) membrane using Towbin standard buffer. Blots were blocked with 5% nonfat dried milk in PBS containing 0.1% Tween-20 and incubated with a mouse monoclonal IgG (C-4: sc-390407, Santa Cruz Biotechnology, Dallas, TX, USA) as primary antibody of GALE, and Alexa-Fluor^TM^ 594 Donkey anti-mouse IgG as a secondary (A-21203, Thermo Fisher Scientific, Waltham, MA, USA) at room temperature. Washed blots were incubated with SuperSignal West Femto solution and exposed to X–ray films. Prior to Western blotting, the gel was treated with Coomassie Blue (Colloidal Blue Staining Kit, Thermo Fisher Scientific, Waltham, MA, USA) for protein staining, washed and used for Western blotting. The same blot was also used for glycans staining with the Schiff base pararosaniline (Schiff′s reagent for aldehydes, Millipore Sigma, St. Louis, MO, USA).

### 2.8. GBM Cell Culture and Sample Preparation for ^13^C and ^1^H NMR Experiments

Cell culture experiments were performed following a similar protocol as reported earlier [5]. Briefly, GMB175 cells (test and control sets; *n* = 3 each) were grown in DMEM, supplemented with 10% FBS, L-glutamine and pyruvate. When cells reached confluency (~80%), cell growth media was replaced with DMEM (Catalog number A1443001, Gibco^TM^; supplemented with 10% FBS, L-glutamine and pyruvate) containing 6.0 mM ^13^C-labelled D-glucose, [U-^13^C]Glc, and the test set was treated with 100 µM 4DFG and incubated for 5 h at 37 °C under humidified air and 5% CO_2_. Finally, the cells were washed with PBS and trypsinized; the resulting cell pellets from both test and control sets were snap-frozen in liquid N_2_ and stored in a −80 °C freezer until further analysis. Cell pellets were extracted in 5% perchloric acid, neutralized with 1.0 M KOH and centrifuged, and the supernatants were dried under vacuum. The residue obtained from each sample was reconstituted in 180 µL D_2_O containing 1.0 mM DSS-d_6_ (as an internal standard), and the pH of the solution was adjusted to 7.4 ± 0.05. ^1^H and ^13^C NMR data were collected on a Bruker NMR spectrometer (Bruker Biospin, Billerica, MA, USA) operating at 800 MHz (^1^H frequency) equipped with a cryogenically cooled ^13^C direct detection probe. A power-gated sequence with a WALTZ-16 composite pulse (flip-angle = 30°) was used to acquire ^1^H-decoupled ^13^C NMR data [5]. Peak areas of ^13^C signals were measured by deconvolution using ACD software version 12.01 (Advanced Chemistry Development, Toronto, ON, Canada). ^13^C NMR isotopomer analysis was performed as reported earlier [5,25]. Briefly, [U-^13^C]Glc is converted to [U-^13^C]pyruvate, via glycolysis, which in turn is converted to [U-^13^C]lactate by lactate dehydrogenase (LDH) (lactate C3 signal at 20.8 ppm, see results Section 3.3 for more details). Further, [U-^13^C]pyruvate is also converted to [1,2-^13^C]acetyl-CoA by pyruvate dehydrogenase (PDH) which enters the tricarboxylic acid (TCA) cycle (in mitochondria) and undergoes further oxidation. In the first turn of the TCA cycle, [1,2-^13^C]acetyl-CoA combines with unlabeled oxaloacetate (OAA) forming [4,5-^13^C]α-ketoglutarate (α-KG) which is in equilibrium with [4,5-^13^C]glutamate (Glu) (glutamate C4 signal at 34.19 ppm, see results Section 3.3 for more details). ^13^C-labeled lactate C3 and glutamate C4 were used for ^13^C isotopomer analysis as reported earlier [5,25].

### 2.9. Glycan Profiling Using Lectin Labeling

An FITC-labeled lectin kit was used to visualize and quantify an array of glycoproteins and glycolipids (EY Laboratories, San Mateo, CA, USA). The binding affinity of FITC lectins is low, with the first-order rate constant (K_off_) of many of the 11 lectins used in the order of 12–15 h. As a result, the images were obtained rapidly. We labeled fixed GBM cells with FITC-lectin suspension for 1 h, washed and then obtained images (*n* = 8 images, of all four treatments: NHA control, GBM vehicle, GBM treated with 4DFG for either 24 or 48 h). All 32 images used in statistical analysis were obtained within a period of 2 h, after the FITC-lectin incubation/wash step.

The primary GBM cell culture GBM175, which had less sensitivity toward the toxic effects of 4DFG than GBM157, was used to examine the 4DFG-induced alterations in glycan synthesis. We used NHA as an internal control for ‘normal’ brain cell glycan profiling. GBM175 cultures were treated with 100 µM 4DFG for either 24 or 48 h, in parallel with NHAs as a normal brain control. At the end of the incubation period, cells were fixed with paraformaldehyde, detergent permeabilized, washed and then treated with DAPI (to label DNA blue) and with FITC lectins. Cell numbers and FITC-lectin signals were quantified to allow the calculation of FITC/cell (*n* = 8). The levels of lectin/cell were then compared in 4 groups: NHAs, Control GBM175 and GBM175 cultures incubated with 100 µM 4DFG for either 24 or 48 h.

### 2.10. Flank and Intracranial Models

All experiments were conducted following the Institutional Animal Care and Use Committee (IACUC) protocol approved by the Houston Methodist Hospital Research Institute (Protocol# IS00004191). GBM xenograft flank tumors were restored from frozen stocks and passaged in nude mice (NU-Foxn1Nu) twice and donor tumors were harvested when the volume was approximately 1.5 cm^3^. Tumors were homogenized using a tissue homogenizer (BeadBug™, Benchmark Scientific, Edison, NJ, USA) in an equal volume of PBS and then diluted with Matrigel™ in 1:1 ratio.

Mice (NU-Foxn1Nu, all female) were given flank xenografts with 150 µL subcutaneous injections in the right flank. Animals were monitored for changes in body weight and tumor volume based on caliper measurements. The greatest longitudinal diameter (length) and greatest transverse diameter (width) were recorded. Volume was calculated by the modified ellipsoidal formula as tumor volume ½ (length × width^2^) [26]. Mice with an average tumor volume of 45 mm^3^ after four weeks were divided into two groups (*n* = 5 in each group). The mice in the two groups were treated three times with either 100 µL saline or 4DFG (2 mg/mL dissolved in saline) injected into the tail vein over the course of a week (Monday/Wednesday/Friday).

Mice (NU-Foxn1Nu, all female) were also given intracranial xenografts with 5 µL injections of GBM157 tumor: Matrigel suspension into the right frontal lobe, via the postglenoid foramen, as previously described [27,28]. The control group consisted of six mice (*n* = 8), whereas treatment group had seven mice (*n* = 8). They were treated six times, over two weeks, with either 100 µL saline (vehicle) or 4DFG (6.25 mg/mL in saline) through the tail vein injection, M/W/F, beginning on day 17, post-xenograft. Animals were monitored for changes in body weight until they reached an ethical endpoint. The survival curves were analyzed using the log-rank method, using GraphPad Prism 9.0 software.

### 2.11. Statistical Analysis

As multiple comparisons were made, we applied the Bonferroni correction in statistical analysis. The Bonferroni corrected *p* = value is equal to *p*-value of an individual *t*-test after being multiplied by the number of comparisons. Thus, in case where NHA signal levels are compared with three different GBM175 treatment groups, the individual *t*-test results are corrected by multiplying by *p*-value by 3 [29]. In the flank and intracranial models, Student’s *t*-test was used to compare the tumor volumes between the control and 4DFG-treated groups, and a *p*-value ≤ 0.05 was considered significant.

## 3. Results

### 3.1. Upregulation of Glut3, GALK1 and GALE in Glioma Tumors

**Figure 2:**
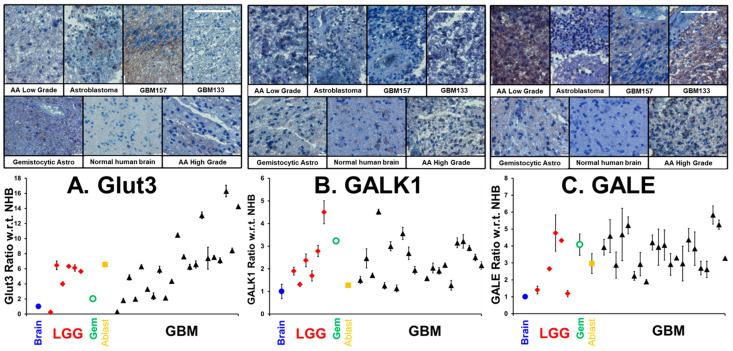
Levels of Glut3, GALK11 and GALE in low grade gliomas (LGGs), gemistocytic glioma (Gem), astroblastoma (Ablast), and GBMs compared to normal human brain. Figure 2 (upper panel) presents selections of a hematoxylin/DAB-stained microarray of immunohistochemically labeled tumors, taken at a magnification of ×20. The white scale bars in the GBM133 samples are 50 µm in length. The lower panel shows the levels of (*n* = 3, mean ± SD,) the enzymes Glut3, GALK1 and GALE in various gliomas compared to the normal brain tissue.

In our previous publication, drawing on three GBM transcriptome databases, we showed that the transcript levels of Glut3/Glut14 and Leloir enzymes were elevated in GBM, and that high transcript levels were statistically correlated with poor patient outcomes [5]. We employed a patient glioma tumor tissue microarray, coupled with immunohistochemistry, to examine the levels of transporters and enzymes required for galactose scavenging proteins. The same tumor microarray and methodologies were employed to quantify antigen levels as described in our earlier investigation showing high expression of MAOB and transcription factors in human gliomas [24]. In addition to a sample of a normal human brain, the microarray contains 6 low-grade gliomas, a gemistocytic glioma, an astroblastoma and 21 GBM tumor samples. In Figure 2 (upper), we show representative images of immunohistochemical labeling of (A) Glut3, (B) GALK1 and (C) GALE, in representative low/high-grade gliomas and the human brain control, visualized with brown-DAB and hematoxylin counter-stained nuclei. Figure 2 (lower panel) shows the mean ± SD levels of (A) Glut3, (B) GALK1 and (C) GALE in gliomas with respect to the normal human brain (*n* = 3). In GBM, the mean levels of Glut3, GALK1 and GALE were elevated (Glut3: 6.6 ± 4.0; GALK1: 2.3 ± 0.9 and GALE: 3.7 ± 1.0) compared to the normal human brain and other gliomas. In Supplementary Figure S1, we show additional pairs of representative images of Glut14 and the average levels of Glut3/Glut14, UDP-glucose dehydrogenase (UGDH), which converts UDP-Glc to UDP-GlcA, and SLC35S2, which is the nucleotide sugar antiporter transporting glycan precursors from the cytosol into the lumen of the Golgi apparatus.

### 3.2. Effects of 4DFG on Growth, GALE and Mitochondria

**Figure 3:**
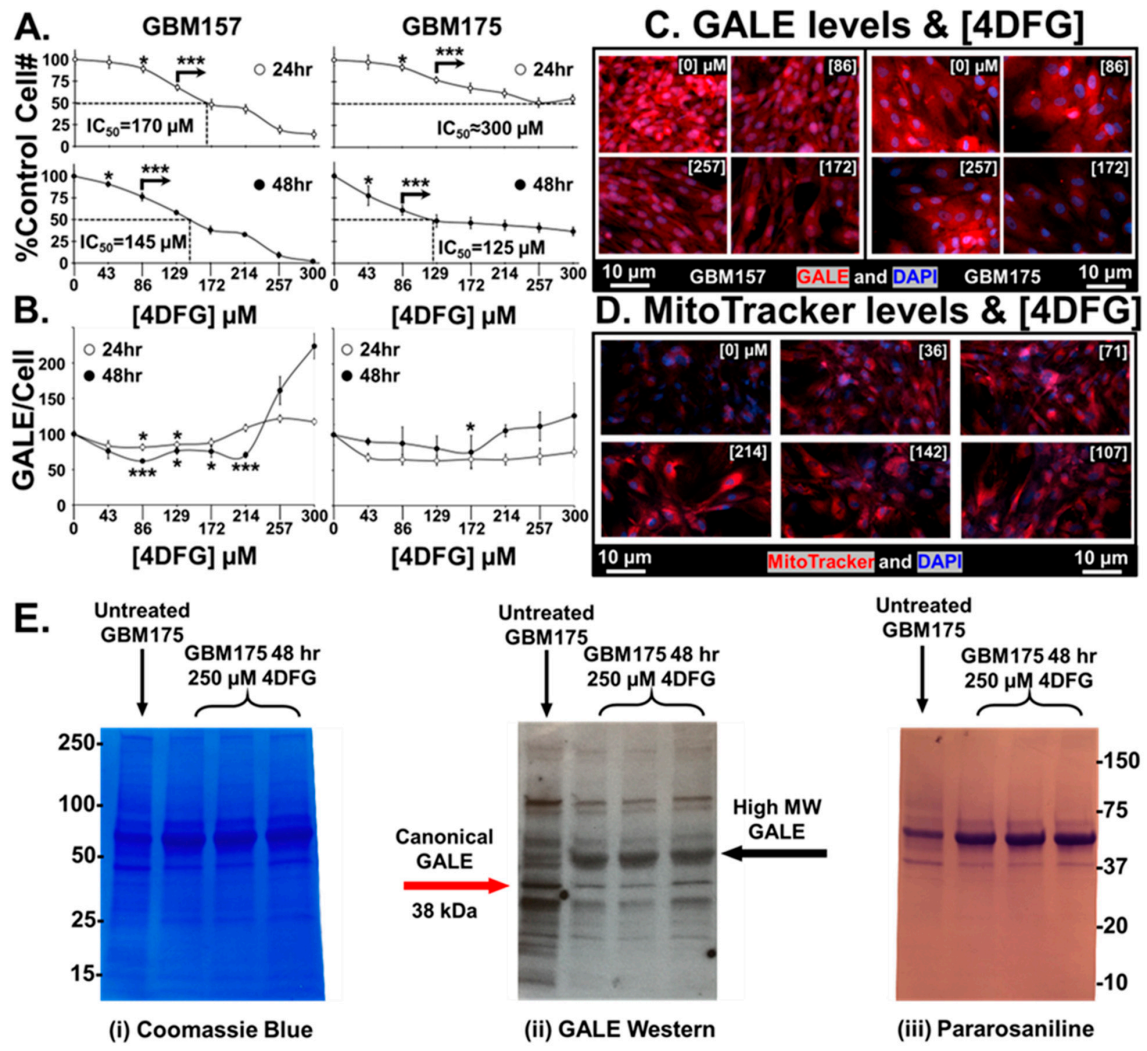
Effects of 4DFG on GBM cells in culture at 24 and 48 h. (**A**) 4DFG causes a concentration and time-dependent drop in living cell number in primary human GBM cells (GBM157 and GBM175) (*n* = 6, mean ± SD). (**B**) The response of GALE levels per cell is dependent on both 4DFG concentration and on incubation time. A *t*-test was used to measure statistical significance (*n* = 4, mean ± SD). It is worth noting that the GALE concentration is increased at higher concentrations of 4DFG (214–300 µM). (**C**) Representative images of GALE in 4DFG-treated GBM157 and GBM175 cells at different 4DFG concentrations (0, 86, 172 and 257 µM). (Magnification: 20×; scale bar: 10 µm) (**D**) Representative images showing increased MitoTracker signals in GBM175 cells at different concentrations of 4DFG (0, 36, 71, 107, 142 and 214 µM). (Magnification: 20×; scale bar: 10 µm) (**E**) GBM175 cells were incubated for 48 h with and without 4DFG (250 μM) and then run on a denaturing gel. The Coomassie stain (on the left) shows that the control cells have similar total protein levels as the 4DFG triplicates. Using an anti-GALE antibody (center) reveals that the control cells have higher levels of the canonical GALE band, ~38 kDa. The 4DFG-treated cells have lower levels of the normal molecular weight GALE band, but has an intense band at ~50 kDa. The gel labeled with the Schiff base pararosaniline (on the right) shows the presence of glycans with terminal reducing sugars. *t*-test with Bonferroni correction; *p* ≤ 0.05, *; and *p* ≤ 0.001, ***.

In Figure 3A, we show that 4DFG causes a concentration and time-dependent drop in living cell numbers in a pair of primary human GBM cells, GBM157 and GBM175, grown in culture (*n* = 6, mean ± SD), for either 24 or 48 h.

A statistically significant drop in cell numbers, at *p* < 0.05, was attained in both cell lines at 24 as well as 48 h, at concentrations of ≥120 µM 4DFG. When incubated for 48 h, the cell number dropped by 50% at concentrations of ~145 µM (IC_50_ = ~145 µM), suggesting that GBM157 cells are clearly more sensitive to the toxic effects of 4DFG than GBM175 cells (IC_50_ = ~125 µM).

Figure 3B shows the levels of GALE/cell (measured by the quantitation of GALE fluorescence intensity) in both GBM157 and GBM175 cultures. GALE levels at <100 µM 4DFG fall initially, and then rise again during the titration between 214–300 µM (*n* = 4, mean ± SD). In our earlier study, we found that GALE levels increase as a function of increased Gal concentration [5]; as a result, there may be an apparent increase in GALE levels when the concentration of the Gal-antimetabolite, 4DFG, goes up. Representative micrographs of GALE staining in both cell lines are shown in Figure 3C. We can clearly see an initial decrease in the GALE immunofluorescence as a function of 4DFG concentration (86 and 172 µM), but it rises at a higher concentration of 4DFG (257 µM).

Figure 3D shows the changes that occur in mitochondrial levels using MitoTracker dye. 4DFG clearly causes an increase in the levels of MitoTracker dye accumulation, indicating that the levels of mitochondria per cell rise in response to exposure to 4DFG. In our earlier study, we have also observed an increased MitoTracker signal in galactose-only incubations in GBM cells [5]. In the current study, the Gal antimetabolite 4DFG may be showing a similar effect.

Figure 3E shows a blot analysis of GALE levels in 4DFG-treated GBM cells (in triplicate), analyzed by three different methodologies. GBM175 cells were incubated for 48 h with and without 4DFG (250 μM) and then run on a denaturing gel. Figure 3E(i) shows the Coomassie staining of proteins in GBM175, and the protein levels in the control (*n* = 1) and 4DFG-treated (*n* = 3) groups were the same. In the center image, Figure 3E(ii), a Western blot analysis showed that there is a ~45% drop in the canonical GALE band at ~38 kDa in the 4DFG-treated group (Supplementary Table S1). However, total GALE labeling was increased by 66% when the additional higher molecular weight bands at ~50–60 kDa were examined (Supplementary Table S2), which is consistent with the cell imaging studies where we observed an increase in GALE at higher concentrations of 4DFG (Figure 3B,C). The cellular protein was also probed using the aldehyde reactive dye, pararosaniline, which showed the presence of glycans with terminal reducing sugars; Figure 3E(iii).

### 3.3. The Effect of 4DFG on ^13^C-Glc Carbon Flux

**Figure 4:**
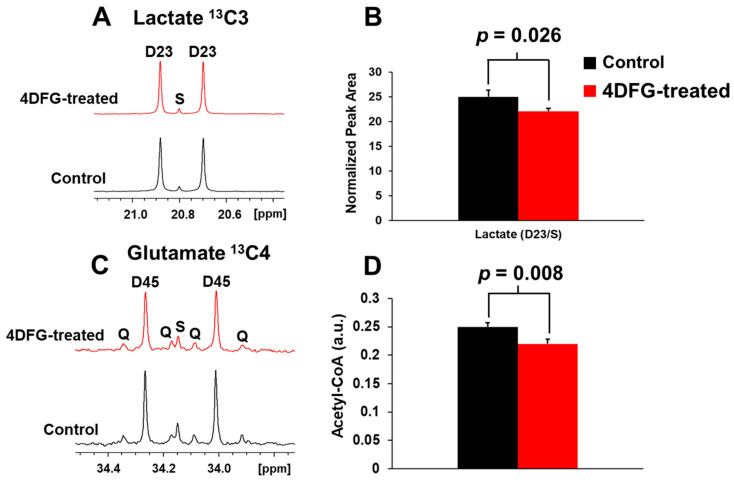
The effect of 4DFG on Glc metabolism in cultured GBM175 cells. (**A**) The NMR spectra of [U-^13^C]lactate derived from [U-^13^C]Glc showing ^13^C3 lactate carbons in the control and 4DFG (100 µM)-treated cells. The doublet D23 arises from ^13^C-^13^C coupling between C2 and C3 carbons of [U-^13^C]lactate, whereas the singlet (S) is due to the natural abundance of C3 carbon of lactate. (**B**) Treatment of GBM cells with 4DFG caused a 11.48 ± 7.27% drop in the lactate synthesis (*n* = 3; statistically significant, Student’s *t*-test, *p* = 0.026), measured using ^13^C NMR isotopomer analysis of ^13^C3 lactate. (**C**) The NMR spectra of [4,5-^13^C]glutamate derived from [U-^13^C]Glc showing ^13^C4 carbons of glutamate in the control and 4DFG-treated cells. [4,5-^13^C]glutamate provides direct measurement of [1,2-^13^C]acetyl-CoA. (**D**) Treatment of GBM cells with 4DFG also caused a 12.06 ± 0.94% reduction in the levels of acetyl-CoA (*n* = 3; statistically significant, Student’s *t*-test, *p* = 0.008) entering the Krebs/TCA cycle.

We wanted to test if 4DFG treatment has any effect on the metabolism of GBM cells (GBM175), and we incubated GBM175 cells with 6 mM [U-^13^C]Glc and 100 μM 4DFG for five hours, and then examined the incorporation of the ^13^C labeling into lactate and glutamate, which are readouts of flux through glycolysis and the Krebs (TCA) cycle, respectively [25]. In Figure 4A, we show the representative NMR spectral regions of the C3-carbon of ^13^C-lactate generated (via glycolysis) from the metabolism of [U-^13^C]Glc in the presence and absence of 4DFG. The doublet (D23) to singlet (S) ratio (D23/S) of ^13^C-lactate provides the relative flux of Glc through glycolysis in GBM175 cells in the presence and absence of 4DFG. There is a statistically significant decrease (by 11.48 ± 7.27%, *p* = 0.026) in the glycolytic flux in 4DFG-treated cells; Figure 4B.

The entry of [U-^13^C]Glc-derived acetyl-CoA into the Krebs cycle was determined by examining the relative levels of [4,5-^13^C]glutamate (C4 carbon) in cells treated with 4DFG compared to the control group; Figure 4C. Again, treatment of GBM cells with 4DFG caused a reduction in mitochondrial carbon flux (by 12.06 ± 0.94%, *p* = 0.008; Figure 4D). The numerical data of glycolytic and mitochondrial flux analysis have been provided in the Supplementary Table S3. The decrease in both the glycolytic and mitochondrial fluxes indicates that 4DFG or its metabolites are inhibiting the generation of pyruvate not only from glycolysis, but also from the pentose phosphate pathway (PPP), which we have previously shown to be highly active in the GBM cell culture [5].

### 3.4. The Effect of 4DFG on Glycan Profiling Using Lectin Labeling

**Figure 5:**
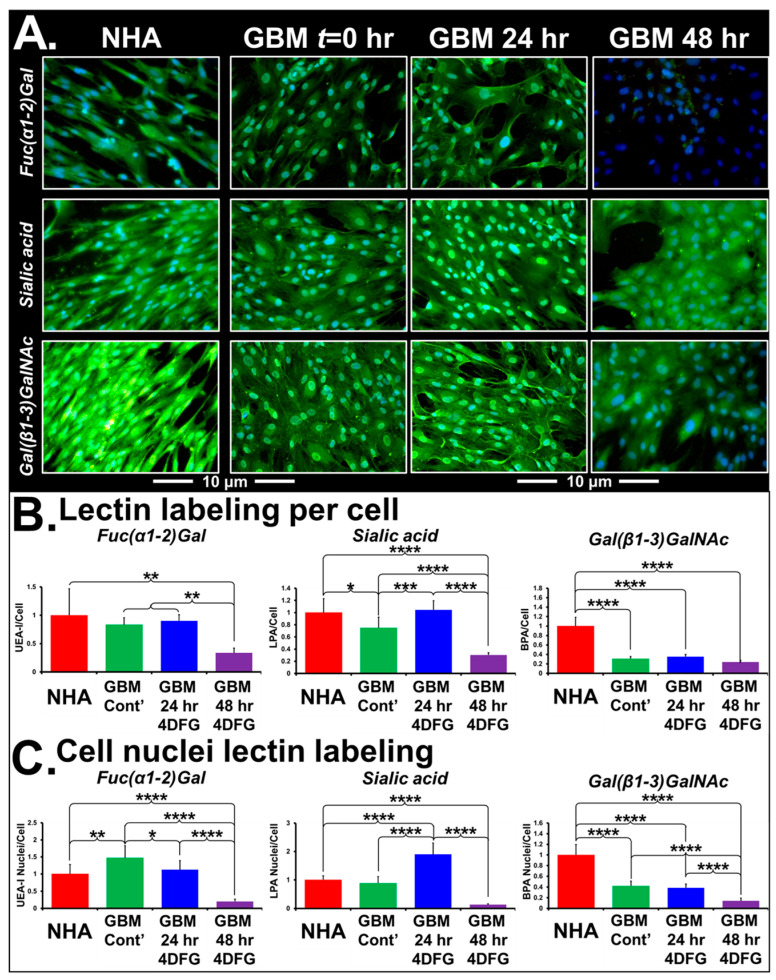
Lectin binding of cells treated with 4DFG shows an alteration in glycan levels in GBM cells. (**A**) shows representative images of NHA and GBM175 cells without (*t* = 0) and with 100 µM 4DFG for 24 h or for 48 h. Green fluorescence indicates levels of three glycans: Fuc(α1-2)Gal (upper panels), Sialic acid (middle panels) or Gal(β1-3)GlcNAc (lower panels). NHA have more Gal(β1-3)GlcNAc than GBM and 4DFG-treated GBM (48 h) have very low Fuc(α1-2)Gal (Magnification: 20×; scale bar: 10 µm). (**B**) shows statistical analysis of lectin labeling/cell, averaged from *n* = 8 wells. (**C**) shows statistical analysis of lectin labeling/cell nucleus, obtained by interrogating 8 individual nuclei in each of the *n* = 8 wells (a total of *n* = 64). *t*-Test with Bonferroni correction; *p* ≤ 0.05, *; *p* ≤ 0.01, **; *p* ≤ 0.001, ***; and *p* ≤ 0.0001, ****.

Figure 5A shows the representative micrographs of NHA and GBM175 cells treated with 100 µM 4DFG for 24 and/or 48 h. The levels of green signal indicate the extent of lectin binding to Fuc(α1-2)Gal, Sialic acid or Gal(β1-3)GlcNAc, with nuclei labeled with DAPI (blue color). Note that the control NHA cells have approximately four times the levels of Gal(β1-3)GlcNAc (labeled by FITC-BPA). In Figure 5B, bar graphs present the levels of lectin/cell with statistical analysis (*n* = 8). In Figure 5C, we have provided the levels of lectin in the cell nucleus with statistical analysis (*n* = 64). Using Sony NIS-elements software, the areas of Hoechst-labeled nuclei (from eight regions/well) were measured. Eight wells were included for each of the control and 4DFG-treated groups, making a total of sixty-four measurements (*n* = 64). The average area of nuclei was ~21% larger in the 4DFG-treated cells at 48 h, (*p* < 0.00001). To validate the authentic lectin binding to glycoproteins, we performed Western blotting of GBM175 cells treated with 100 µM 4DFG for 24 h and visualized using an anti-FITC antibody to the chosen FITC lectins. In Supplementary Figure S2, we show that the treatment of GBM175 with 4DFG alters the pattern of lectin labeling of cell lysates, especially of the lectins PNA and MPA, which can both label Gal(β1-3)GalNAc-containing glycans.

In Table 1, we present the analysis of changes in the labeling of whole detergent permeabilized cells and the nuclear localization of six lectins: LPA (Neu5Ac (sialic acid)), UEA-I (Fuc(α1-2)Gal), SBA (α-/β- GalNAc), BPA (Gal(β1-3)GlcNAc), MPA (Gal(β1-3)GalNAc and GalNAc(α1-6)Gal) and WGA(GlcNAc(β1-4)GlcNAc). The glycan specificity of the lectins used in the study and their parental species are shown in Supplementary Table S4. Incubation of cells with 4DFG completely alters the levels of glioma glycans in a time-dependent manner. The signal levels of all of these six FITC lectins from NHA are highly responsive to 4DFG-derived metabolites acting as inhibitors of GALE and UGDH. Except for LPA bound to sialic acid, after 24 h of 4DFG treatment, we observed a drop in the nuclear localization of lectin binding, with respect to total cellular glycan levels, with a further decrease in the lectin binding at 48 h. Examining LPA-bound sialic acid, we observed an increase in terminal sialic acid glycan ends directed towards glycans targeted to the nucleus at 24 h, followed by a collapse at 48 h. This suggests that 4DFG-derived metabolites cause inhibition of GALE and UGDH, subsequently leading to low levels of UDP-Gal/UDG-glucoronate, which are the glycan substrates of sialyltransferases. Increasing the incubation time to 48 h decreased the nuclear/total signal ratio for all FITC-lectin bindings (including those listed in Supplementary Table S4, but not listed in Table 1 (GSL-I, GSL-II, PNA, DBA and Con A).

### 3.5. The Effect of 4DFG in Primary GBM Xenografts in Nude Mice

**Figure 6:**
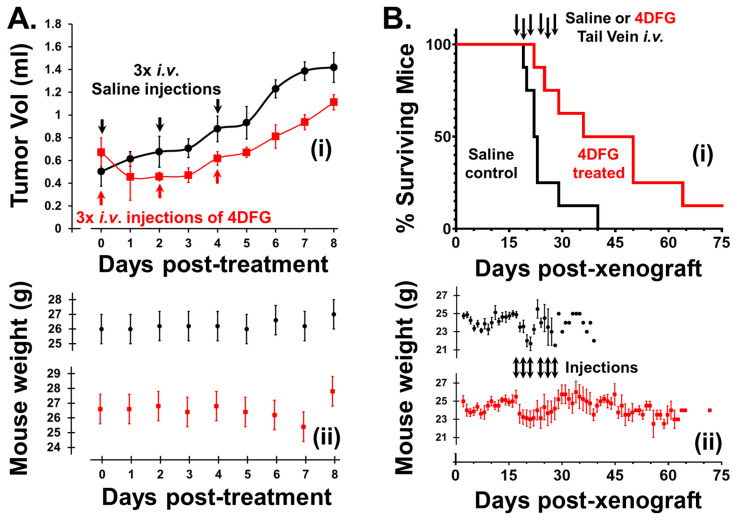
Effects of 4DFG on GBM xenografts in nude mice (Black data points/lines: Saline treated (vehicle); and Red data points/lines: 4DFG treated). (**A**) The effect of 4DFG (8 mg/kg) in mice with GBM flank tumors after three tail vein injections, two days apart. After the first 4DFG injection, all treated tumors showed shrinkage. Analysis of volume changes showed a 30% drop in tumor growth over 8 days of observation (*t*-test, *p* < 0.01, *n* = 5). The tumor volume remained static after the second and third treatments, and tumor growth was only observed 48 h after the last injection (**i**). The weights of the animals in the two groups do not exhibit any large changes after the injections of either saline or 4DFG in saline (**ii**). (**B**) Survival curves of mice with intracranial GBM157 xenografts treated with 4DFG (**i**). Mice that were treated with 4DFG (25 mg/kg) three times a week for two weeks performed significantly better (*t*-test, *p* = 0.01, *n* = 8 in control group; *n* = 8 in treated group) than the control group, with their survival time doubling (**i**). In mice with intracranial tumors, the animals lost weight following tail vein injections, but the decline was observed in both groups (**ii**).

We examined the effect of 4DFG (8 mg/kg) on primary GBM flank tumor volume in nude mice. The blood volume of a 25 g mouse is approximately 1.4 mL and this treatment gives rise to a serum 4DFG concentration of ~800 µM, which is more than the concentration of 4DFG used in continuous incubation studies (100–300 µM), but less than the ~1 mM, Km of GALK1 for Gal [30]. This low level of 4DFG was used in the first animal study to mainly explore 4DFG toxicity in mice, and this was the concentration initially approved by our IACUC committee. In Figure 6A, the upper plot (i) shows the change in the primary GBM tumor volume in animals that received either a saline (vehicle) or 4DFG injection to the tail vein (*n* = 5 in each group), the lower plot (ii) shows the weights of mice recorded at different time intervals.

4DFG halts the growth in primary human GBM tumors in mouse flanks. After the first injection (Day 0), all 4DFG-treated tumors showed shrinkage. The tumor volume remained static after the second (Day 2) and third (Day 4) injections, and tumor growth was observed 48 h after the last injection (Day 6). Analysis of volume changes indicated a 30% drop in tumor growth over 8 days of observation (*t*-test, *p* < 0.01, *n* = 5). Additionally, the lesions which were observable on the skin of the nude mice above the tumor lost their redness and they either shrunk or disappeared in the 4DFG treatment group. Two of the control animals reached an ethical endpoint of 8 days due to ulceration of the skin above the tumor. The weights of the animals in the two groups did not exhibit any large changes after the injections in either saline or 4DFG treated groups.

### 3.6. The Effect of 4DFG in Intracranial Primary GBM Xenograft Model

In Figure 6B, we show survival curves of mice with intracranial GBM157 xenografts treated with 4DFG. The animals were treated with vehicle (*n* = 8) on days 17, 19, 21, 24, 26 and 28, post-xenograft, with 100 µL tail vein injections of saline or 4DFG dissolved in saline. Since we observed no associated toxicity in the flank model study, the mice were treated with a higher dose of 4DFG (25 mg/kg) in the intracranial model. In the 4DFG (25 mg/kg)-treated group (*n* = 8), the post-injection peak concentration of the drug should be near 2.5 mM, a concentration in the physiological range of serum Gal, and at this concentration, they will be able to saturate the galactose-scavenging enzymes of the glioma cells.

The vehicle-control cohort reached an ethical end point at a median time of 22 days. The treated mice performed significantly better (*t*-test, *p* < 0.01) with a median time of 44 days. One mouse treated with 4DFG was healthy and symptom-free for more than one year after treatment. We observed no symptoms of 4DFG-induced toxicity during the study and the weights of both cohorts underwent a similar drop in the injection phase of the study; Figure 6B(ii).

## 4. Discussion

4DFG is designed to be converted into UDP-4DFG, a potent competitive inhibitor of GALE (in the low 10s of µM range) at physiologic concentrations of UDP-Gal/UDP-Glc (100–300 nM) [31,32,33]. 4DFG can be converted into UDP-4DFG via the sequential action of Glut3/14, GALK1 and GALT. GALK1 can phosphorylate 4DFG, which is a substrate for GALT, for the synthesis of UDP-4DFG [32,34,35]. UDP-4DFG is also a potent inhibitor of UGDH, (K_i_ of ≈20 µM); inhibiting UGDH and results in the saturation of UDP-Gal/UDP-Glc levels. 4DFG and similar Gal-based anti-metabolite chemotherapeutics can potentially have differential toxicity toward cancer cells if Glut3/14 and the Leloir cycle enzymes are elevated in cancer cells [36,37,38,39,40,41].

Our current study demonstrates that patient-derived GBM tumors have high expression of Glut3/14 and of Leloir pathway enzymes. We have shown that the Gal-based antimetabolite 4DFG is toxic toward GBM cells in culture and in mouse xenograft models. Metabolic flux analysis using ^13^C-Glc (as a tracer) in the presence and absence of 4DFG in patient-derived GBM cells revealed a decrease in both glycolytic and mitochondrial fluxes, which indicates that 4DFG and its metabolites are inhibiting the generation of pyruvate from glycolysis and from the pentose phosphate pathway, both of which we have previously shown to be highly active in GBM cell cultures [5].

Our glycan profiling studies demonstrate that treatment of GBM cells with 4DFG completely alters their glycan profile. We observed a noteworthy alteration in the location of glycan binding following 4DFG incubation, which we believe represents alterations in the levels of Gal- and glucuronate-containing glycan turnover in two main pools, glycolipids (which have a slow turnover) and glycoproteins (which have a relatively high turnover). The observed collapse of the ratio of nuclear vs. whole cell FITC-lectin signaling probably reflects the relative turnover times of the two major glycan pools, glycolipids and proteoglycans.

After treatment with 4DFG, gliomas compensate for the low levels of glycan building blocks (UDP-Glc, UDP-Gal and UDP-GlcA) by increasing sugars that derive from the hexamine pathway, as indicated by the nature of the glycans observed after 4DFG treatment, which utilize reducing sugar terminals and are more heavily fucosylated, and utilize terminal GlcNAc, GalNAc and sialyl ends.

In our in vivo mouse model studies, we observed a modest impact on GBM tumor growth (~30% decrease in the tumor volume) in an initial flank tumor study, where we used a low concentration of 4DFG. In the intracranial model of GBM, we observed a large increase in animal survival after 4DFG treatment. Although we do not envisage 4DFG being used as a monotherapeutic agent in human patients, Gal-based antimetabolites should complement existing standard of care (surgery followed by chemoradiotherapy) in the treatment of GBM tumors.

Although we were able to see the inhibitory effect of 4DFG on patient-derived GBM cells, our in vivo studies had some limitations. In the flank mouse model, we were unable to wait for a longer time and see the actual shrinkage of tumors as a few animals developed ulceration. In the intracranial model, we observed a significant (*p* < 0.01) increase in the overall survival in the 4DFG-treated mice. However, we were unable to obtain tumor shrinkage data by MRI. In future studies, we will use an optimal dose of 4DFG and use imaging techniques such as MRI to monitor tumor size during the drug treatment period in both the flank and intracranial models. Furthermore, we will harvest the tumors at the end of treatment period and perform immunohistochemical characterization (H&E and Ki-67) of tumor tissues and use this information to differentiate between the treated and control groups.

## 5. Conclusions

GBM cells are unique in their expression of Glut3/14, allowing efficient Gal import and metabolism via the Leloir pathway; as a result, Gal-based antimetabolites will have a differential impact on GBM cells and will have potential in GBM therapy. Our data suggest that glycan synthesis in GBM is a valid target for chemotherapy using Gal-based antimetabolites such as 4DFG.

## Figures and Tables

**Table 1:** Alterations in levels and localization of glioma glycans following incubation with 100 µM 4DFG. Statistical analysis of alterations in lectin binding to glycans and their localization within glioma following incubation with 100 µM 4DFG for 24 or 48 h. It is noticeable that at 48 h there is a large drop in all glycans, but at 24 h, due to remodeling, some glycan terminal end types (i.e., sialyl) increase with respect to control.

Lectin	NHA = 100%	GBM175 *t* = 0	4DFG 24 h	4DFG 48 h
LPA Neu5Ac (sialic acid)	Signal/Cell	75%	104.6%	30.4%
	*p* w.r.t. GBM175		0.003	9.9 × 10^−5^
	Nuclear Signal	89.1%	190.2%	13.4%
	*p* w.r.t. GBM175		2 × 10^−5^	1.6 × 10^−7^
UEA-I Fuc(α1-2) (Gal)	Signal/Cell	83.3%	90.3%	33.7%
	*p* w.r.t. GBM175		0.25	0.011
	Nuclear Signal	147.1%	112.6%	20.2%
	*p* w.r.t. GBM175		0.045	1.2 × 10^−7^
SBA α-/β- GalNAc > α-/β- Gal	Signal/Cell	20.8%	34.6%	16.4%
	*p* w.r.t. GBM175		0.00014	0.5
	Nuclear Signal	33.1%	36.1%	14.2%
	*p* w.r.t. GBM175		0.43	6.6 × 10^−6^
BPA Gal(β1-3)-GlcNAc	Signal/Cell	30.9%	35.3%	23.9%
	*p* w.r.t. GBM175		0.07	0.32
	Nuclear Signal	42.1%	38.7%	14.1%
	*p* w.r.t. GBM175		0.41	2 × 10^−6^
MPA Gal(β1-3)-GalNAc & GalNAc(α1-6)-Gal	Signal/Cell	92.0%	112.3%	91.7%
	*p* w.r.t. GBM175		0.2	0.98
	Nuclear Signal	179.2%	151.1%	53%
	*p* w.r.t. GBM175		0.38	0.00023
WGA GlcNAc(β1-4)-GlcNAc	Signal/Cell	88.73%	83.5%	44.8%
	*p* w.r.t. GBM175		0.63	3 × 10^−7^
	Nuclear Signal	76.1%	67.6%	15%
	*p* w.r.t. GBM175		0.39	6.6 × 10^−7^

## Data Availability

The data presented in this study are available upon request from the corresponding authors.

## Supplementary Materials

The following supporting information can be downloaded at: https://www.mdpi.com/article/10.3390/cancers16203510/s1, Figure S1: Representative images of tumors from the microarray of Glut14, UGDH and SLC35S2. Also shown are derived levels of these proteins with respect to normal human brain. Figure S2: Levels and molecular weight of glycans in GBM175 cells after 4DFG incubation. Table S1: Western blot analysis of low MW (38 kDa) GALE in GBM175 cells with and without the treatment of 4DFG. Table S2: Western blot analysis of high MW (50–60 kDa) GALE in GBM175 cells with and without the treatment of 4DFG. Table S3: Levels of lactate and acetyl-CoA generated from ^13^C-Glc in GBM175 cells in the presence and absence of 4DFG (determined by ^13^C NMR isotopomer analysis). Table S4: Lectins used in this study, with species name, abbreviation and their specificity.

## References

[1] Ostrom Q.T., Price M., Neff C., Cioffi G., Waite K.A., Kruchko C., Barnholtz-Sloan J.S. (2023). CBTRUS Statistical Report: Primary Brain and Other Central Nervous System Tumors Diagnosed in the United States in 2016–2020. Neuro-Oncology.

[2] Lassman A.B., Joanta-Gomez A.E., Pan P.C., Wick W. (2020). Current usage of tumor treating fields for glioblastoma. Neurooncol. Adv..

[3] Parodi A.J. (2012). Luis Federico Leloir, or how to do good science in a hostile environment. Iubmb Life.

[4] Decker D., Kleczkowski L.A. (2019). UDP-Sugar Producing Pyrophosphorylases: Distinct and Essential Enzymes with Overlapping Substrate Specificities, Providing de novo Precursors for Glycosylation Reactions. Front. Plant Sci..

[5] Sharpe M.A., Ijare O.B., Baskin D.S., Baskin A.M., Baskin B.N., Pichumani K. (2021). The Leloir Cycle in Glioblastoma: Galactose Scavenging and Metabolic Remodeling. Cancers.

[6] Sharpe M.A., Baskin D.S., Johnson R.D., Baskin A.M. (2023). Acquisition of Immune Privilege in GBM Tumors: Role of Prostaglandins and Bile Salts. Int. J. Mol. Sci..

[7] Cao J., Zhang Z., Zhou L., Luo M., Li L., Li B., Nice E.C., He W., Zheng S., Huang C. (2023). Oncofetal reprogramming in tumor development and progression: novel insights into cancer therapy. MedComm.

[8] Barzegar Behrooz A., Latifi-Navid H., da Silva Rosa S.C., Swiat M., Wiechec E., Vitorino C., Vitorino R., Jamalpoor Z., Ghavami S. (2023). Integrating Multi-Omics Analysis for Enhanced Diagnosis and Treatment of Glioblastoma: A Comprehensive Data-Driven Approach. Cancers.

[9] Huang R., Li M., Zeng Z., Zhang J., Song D., Hu P., Yan P., Xian S., Zhu X., Chang Z. (2022). The Identification of Prognostic and Metastatic Alternative Splicing in Skin Cutaneous Melanoma. Cancer Control..

[10] Han X., Han B., Luo H., Ling H., Hu X. (2023). Integrated Multi-Omics Profiling of Young Breast Cancer Patients Reveals a Correlation between Galactose Metabolism Pathway and Poor Disease-Free Survival. Cancers.

[11] Colville C.A., Seatter M.J., Jess T.J., Gould G.W., Thomas H.M. (1993). Kinetic analysis of the liver-type (GLUT2) and brain-type (GLUT3) glucose transporters in Xenopus oocytes: Substrate specificities and effects of transport inhibitors. Biochem. J..

[12] Nishimura H., Pallardo F.V., Seidner G.A., Vannucci S., Simpson I.A., Birnbaum M.J. (1993). Kinetics of GLUT1 and GLUT4 glucose transporters expressed in Xenopus oocytes. J. Biol. Chem..

[13] Burant C.F., Bell G.I. (1992). Mammalian facilitative glucose transporters: Evidence for similar substrate recognition sites in functionally monomeric proteins. Biochemistry.

[14] Uldry M., Ibberson M., Hosokawa M., Thorens B. (2002). GLUT2 is a high affinity glucosamine transporter. FEBS Lett..

[15] Deng D., Sun P., Yan C., Ke M., Jiang X., Xiong L., Ren W., Hirata K., Yamamoto M., Fan S. (2015). Molecular basis of ligand recognition and transport by glucose transporters. Nature.

[16] Häuselmann I., Borsig L. (2014). Altered tumor-cell glycosylation promotes metastasis. Front. Oncol..

[17] Holmes E.H., Ostrander G.K., Clausen H., Graem N. (1987). Oncofetal expression of Lex carbohydrate antigens in human colonic adenocarcinomas. Regulation through type 2 core chain synthesis rather than fucosylation. J. Biol. Chem..

[18] Ashkani J., Naidoo K.J. (2016). Glycosyltransferase Gene Expression Profiles Classify Cancer Types and Propose Prognostic Subtypes. Sci. Rep..

[19] Kato Y., Hayatsu N., Kaneko M.K., Ogasawara S., Hamano T., Takahashi S., Nishikawa R., Matsutani M., Mishima K., Narimatsu H. (2008). Increased expression of highly sulfated keratan sulfate synthesized in malignant astrocytic tumors. Biochem. Biophys. Res. Commun..

[20] Cuello H.A., Ferreira G.M., Gulino C.A., Toledo A.G., Segatori V.I., Gabri M.R. (2020). Terminally sialylated and fucosylated complex N-glycans are involved in the malignant behavior of high-grade glioma. Oncotarget.

[21] Dusoswa S.A., Verhoeff J., Abels E., Méndez-Huergo S.P., Croci D.O., Kuijper L.H., de Miguel E., Wouters V.M.C.J., Best M.G., Rodriguez E. (2020). Glioblastomas exploit truncated O-linked glycans for local and distant immune modulation via the macrophage galactose-type lectin. Proc. Natl. Acad. Sci. USA.

[22] Sala-Rabanal M., Hirayama B.A., Ghezzi C., Liu J., Huang S.C., Kepe V., Koepsell H., Yu A., Powell D.R., Thorens B. (2016). Revisiting the physiological roles of SGLTs and GLUTs using positron emission tomography in mice. J. Physiol..

[23] Marcus D.M., Westwood J.H. (1971). Fluorinated carbohydrates. IV. 4-deoxy-4-fluoro-D-galactose. Carbohydr. Res..

[24] Sharpe M.A., Baskin D.S. (2016). Monoamine oxidase B levels are highly expressed in human gliomas and are correlated with the expression of HiF-1α and with transcription factors Sp1 and Sp3. Oncotarget.

[25] Ijare O.B., Baskin D.S., Sharpe M.A., Pichumani K. (2018). Metabolism of fructose in B-cells: A 13C NMR spectroscopy based stable isotope tracer study. Anal. Biochem..

[26] Tomayko M.M., Reynolds C.P. (1989). Determination of subcutaneous tumor size in athymic (nude) mice. Cancer Chemother. Pharmacol..

[27] Raghavan S., Baskin D.S., Sharpe M.A. (2020). MP-Pt(IV): A MAOB-Sensitive Mitochondrial-Specific Prodrug for Treating Glioblastoma. Mol. Cancer Ther..

[28] Iwami K., Momota H., Natsume A., Kinjo S., Nagatani T., Wakabayashi T. (2012). A novel method of intracranial injection via the postglenoid foramen for brain tumor mouse models. J. Neurosurg..

[29] Bland J.M., Altman D.G. (1995). Multiple significance tests: The Bonferroni method. Bmj.

[30] Timson D.J., Reece R.J. (2003). Functional analysis of disease-causing mutations in human galactokinase. Eur. J. Biochem..

[31] Chapeau M.-C., Frey P.A. (1994). Synthesis of UDP-4-deoxy-4-fluoroglucose and UDP-4-deoxy-4-fluorogalactose and their Interactions with Enzymes of Nucleotide Sugar Metabolism. J. Org. Chem..

[32] Timson D.J., Reece R.J. (2003). Sugar recognition by human galactokinase. BMC Biochem..

[33] Lazarowski E.R., Harden T.K. (2015). UDP-Sugars as Extracellular Signaling Molecules: Cellular and Physiologic Consequences of P2Y(14) Receptor Activation. Mol. Pharmacol..

[34] Thomas P., Bessell E.M., Westwood J.H. (1974). The use of deoxyfluoro-D-galactopyranoses in a study of yeast galactokinase specificity. Biochem. J..

[35] Errey J.C., Mukhopadhyay B., Kartha K.R., Field R.A. (2004). Flexible enzymatic and chemo-enzymatic approaches to a broad range of uridine-diphospho-sugars. Chem. Commun..

[36] Cosset E., Ilmjärv S., Dutoit V., Elliott K., von Schalscha T., Camargo M.F., Reiss A., Moroishi T., Seguin L., Gomez G. (2017). Glut3 Addiction Is a Druggable Vulnerability for a Molecularly Defined Subpopulation of Glioblastoma. Cancer Cell.

[37] Labak C.M., Wang P.Y., Arora R., Guda M.R., Asuthkar S., Tsung A.J., Velpula K.K. (2016). Glucose transport: Meeting the metabolic demands of cancer, and applications in glioblastoma treatment. Am. J. Cancer Res..

[38] Masin M., Vazquez J., Rossi S., Groeneveld S., Samson N., Schwalie P.C., Deplancke B., Frawley L.E., Gouttenoire J., Moradpour D. (2014). GLUT3 is induced during epithelial-mesenchymal transition and promotes tumor cell proliferation in non-small cell lung cancer. Cancer Metab..

[39] McKinnon B., Bertschi D., Wotzkow C., Bersinger N.A., Evers J., Mueller M.D. (2014). Glucose transporter expression in eutopic endometrial tissue and ectopic endometriotic lesions. J. Mol. Endocrinol..

[40] Krzeslak A., Wojcik-Krowiranda K., Forma E., Jozwiak P., Romanowicz H., Bienkiewicz A., Brys M. (2012). Expression of GLUT1 and GLUT3 glucose transporters in endometrial and breast cancers. Pathol. Oncol. Res..

[41] Boado R.J., Black K.L., Pardridge W.M. (1994). Gene expression of GLUT3 and GLUT1 glucose transporters in human brain tumors. Brain Res. Mol. Brain Res..

