# Supplementary material for: Targeting the Leloir Pathway with Galactose-Based Antimetabolites in Glioblastoma
Source: Cancers (Basel). 2024 Oct 17;16(20):3510. doi: 10.3390/cancers16203510 (PMC11506710; doi:10.3390/cancers16203510)
Supplement: Supplementary file 1 [file cancers-16-03510-s001.zip › cancers-3205771-supplementary.pdf]

cancers

MDPI

Article

Targeting the Leloir pathway with galactose-based antimetabolites in glioblastoma

Martyn A. Sharpe[1, 2,3*,†], Omkar B. Ijare[1,2,3,4,*,†], Sudhir Raghavan[1,2,3], Alexandra M. Baskin[1,2,3], Brianna N. Baskin[1,2,3], and David S. Baskin[1,2,3,4,5]

[1]Kenneth R. Peak Brain and Pituitary Tumor Treatment Center, Houston Methodist Hospital, Houston, Texas, USA

2 Department of Neurosurgery, Houston Methodist Neurological Institute, Houston Methodist Hospital and Research Institute, Houston, TX, 77030, USA

3 Houston Methodist Academic Institute, Houston, Texas, USA

4 Weill Cornell Medical College, New York, NY, USA

5 Texas A & M Medical School, Houston, Texas USA

* Correspondence: masharpe@houstonmethodist.org; Tel. +1-713-363-6995 (MAS): oijare@houstonmethodist.org; Tel. :+1-713-441-3547 (OBI)

† These authors contributed equally to this work

Supplementary Information:

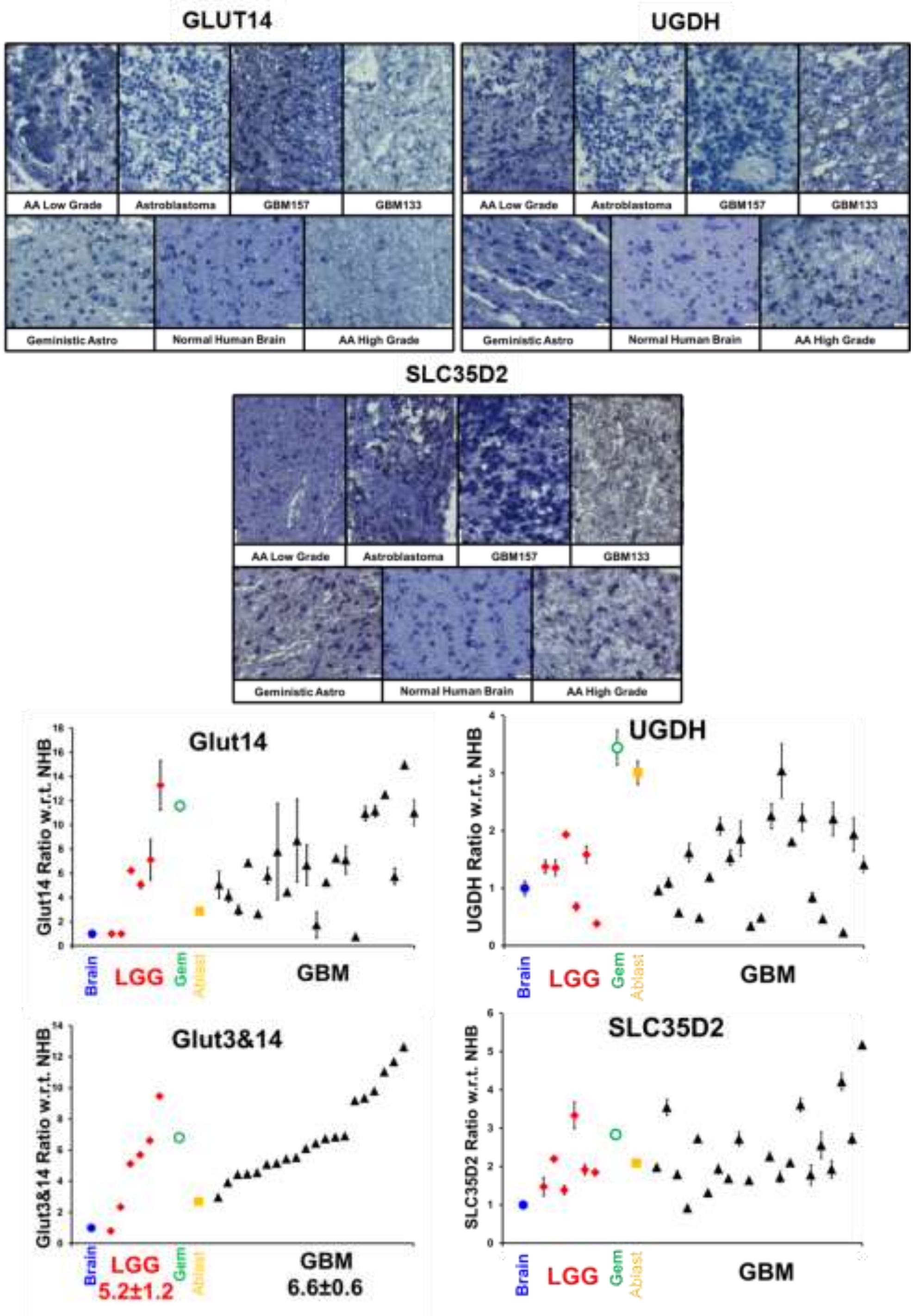

Figure S1. Levels of Glut14, Glut3+Glut14, UGDH and SLC35D2 (A nucleotide sugar transporter involved in the transportation of UDP-sugars from the cytosol into the lumen of the Golgi apparatus) in gliomas compared to normal human brain. Upper and middle panels present selections of a hematoxylin/DAB-stained microarray of immunohistochemically labelled tumors, taken at a magnification of 20x (The white scale bars = 50 µm). Lower panels show levels of these enzymes compared to a normal brain (n=3; Mean ± SD).

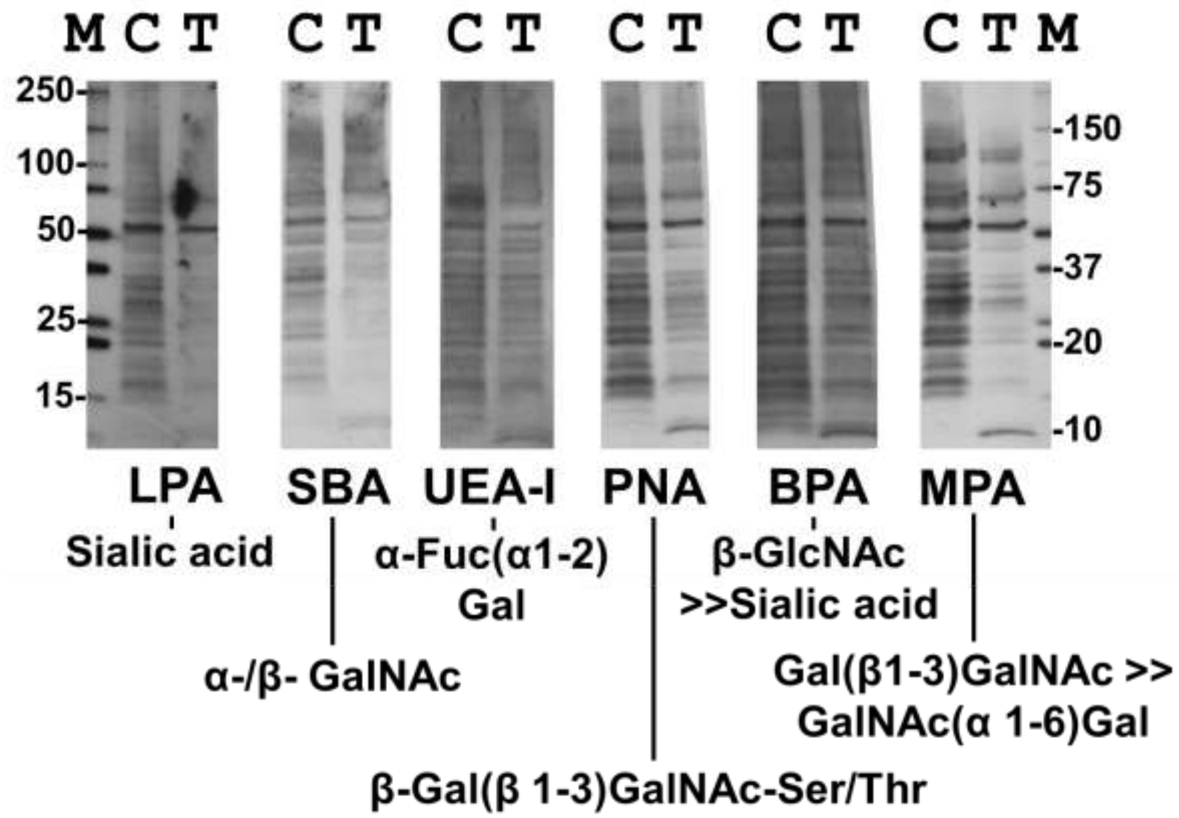

Figure S2. Levels and molecular weight of glycans in GBM175 cells after 4DFG incubation. GBM175 cells were grown with and without 100 µM 4DFG for 24 hrs. Control/4DFG pairs (Control/treated; C/T) were labeled with lectins and developed generating an alteration in the glycan patterns of expression are altered by incubation with 4DFG. The lectin ID and specificity are shown below and the molecular weight to the sides. M: molecular weight.

Table S1: Western Blot analysis of low MW (38 kDa) GALE in GBM175 Cells with and without the treatment of 4DFG.

GBM175 Cells	Band Area*			Mean	**%decrease**
Control (n=1)*	686	714	672	690.6666667	
4DFG-treated (n=3)	400	414	336	383.3333333	**44.4980695**

*Band area was measured 3 times, since number of replicate for control set was one, n=1.

Table S2: Western Blot analysis of high MW (50-60 kDa) GALE in GBM175 Cells with and without the treatment of 4DFG.

**GBM175 Cells**	**Band Area***			**Mean**	**%Increase**
**Control (n=1)***	**598**	**675**	**690**	**654.3333333**	
**4DFG-treated (n=3)**	**1012**	**1100**	**1144**	**1085.333333**	**65.86856852**

*Band area was measured 3 times, since number of replicate for control set was one, n=1.

Table S3: Levels of lactate and acetyl-CoA generated from ^{13}C-Glc in GBM175 cells in the presence and absence of 4DFG (determined by ^{13}C NMR isotopomer analysis).

**C3-Lactate**	**Normalized Peak Area**															
	**CONTROL**					**4-DFG TREATED**			Mean	SD	**P-value**	%change				
Trials	T1	T2	T3	Mean	SD	T1	T2	T3				T1	T2	T3	**Mean**	**SD**
S	0.037426	0.037286	0.040958	3.86E-02	0.002081	0.044299	0.043687	0.042213	4.34E-02	0.001072	0.023	18.36	17.17	3.06	12.87	8.508757
D23	0.962574	0.962714	0.959042	9.61E-01	0.002081	0.955701	0.956313	0.957787	9.57E-01	0.001072	0.023	-0.71	-0.66	-0.13	-0.50	0.323428
**D23/S**	**25.71933**	**25.8198**	**23.41528**	**2.50E+01**	**1.360176**	**21.5741**	**21.89003**	**22.68939**	**2.21E+01**	**0.574844**	**0.026**	**-16.12**	**-15.22**	**-3.10**	**-11.48**	**7.270301**

**C4-Glutamate**	**Normalized Peak Area**															
	**CONTROL**					**4-DFG TREATED**			Mean	SD	P-value	%change				
Trials	T1	T2	T3	Mean	SD	T1	T2	T3				T1	T2	T3	Mean	SD
S	0.098287	0.101321	0.096551	9.87E-02	0.002414	0.0846	0.088387	0.096872	9.00E-02	0.006284	0.087	-13.93	-12.76	0.33	-8.79	7.918269
D45	0.683249	0.683606	0.690507	6.86E-01	0.004091	0.721654	0.71181	0.704062	7.13E-01	0.008817	0.009	5.62	4.13	1.96	3.90	1.839035
Q	0.218463	0.215073	0.212942	2.15E-01	0.002785	0.193745	0.199802	0.199065	1.98E-01	0.003305	0.002	-11.31	-7.10	-6.52	-8.31	2.617848
D45/S	6.951566	6.746937	7.151751	6.95E+00	0.202411	8.530155	8.053295	7.26795	7.95E+00	0.637354	0.061	22.71	19.36	1.62	14.57	11.33089
Q/S	2.222706	2.12269	2.205491	2.18E+00	0.053472	2.290126	2.260524	2.054927	2.20E+00	0.128105	0.831	3.03	6.49	-6.83	0.90	6.911585
**Fc3=(C4/C3)*Q**	**0.243561**	**0.257717**	**0.248614**	**2.50E-01**	**0.007174**	**0.214538**	**0.228856**	**2.16E-01**	**2.20E-01**	**0.007854**	**0.008**	**-11.92**	**-11.20**	**-13.08**	**-12.06**	**0.948353**

Table S4: Lectins used in this study, with species name, abbreviation and their specificity.

	Lectins used in this study		
#	***Lectin Derived Species***	**Abbrev.**	***Specificity***
1	*Limulus polyphemus*	LPA	Neu5Ac (sialic acid)
2	*Glycine max*	SBA	Terminal α-/β- GalNAc > α-/β- Gal
3	*Ulex europaeus*	UEA-I	Fuc(α1-2)Gal
4	*Griffonia simplicifolia*	GSL-I	Terminal α-Gal
5		GSL-II	Terminal α-/β- GlcNAc >> glycogen
6	*Triticum vulgaris*	WGA	GlcNAc(β1-4)GlcNAc(β1-4)GlcNAc >> Neu5Ac (sialic acid)
7	*Arachis hypogaea*	PNA	Gal(β1-3)GalNAc(α1-Ser/Thr) T-Antigen
8	*Bauhinia purpurea*	BPA	Terminal Gal(β1-3)GlcNAc
9	*Dolichos biflorus*	DBA	GalNAc(α1-3)GalNAc
10	*Maclura pomifera*	MPA	Terminal Gal(β1-3)GalNAc > GalNAc(α 1-6)Gal
11	*Canavalia ensiformis*	Con A	Branched α -mannosidic structures
